# Wettbewerb in Zeiten der Pandemie

**DOI:** 10.1007/s10273-020-2785-1

**Published:** 2020-11-20

**Authors:** David Benček, Lorela Ceni-Hulek, Achim Wambach, John Weche

**Affiliations:** 1 Monopolkommission, Heilsbachstr. 17, 53123 Bonn, Deutschland; 2 Monopolkommission, Heilsbachstr. 18, 53123 Bonn, Deutschland; 3 ZEW, L 7, 1, 68161 Mannheim, Deutschland; 4 Monopolkommission, Heilsbachstr. 16, 53123 Bonn, Deutschland

**Keywords:** L1, L4, L5, D4

## Abstract

Zahlreiche Märkte sind durch die Coronavirus-Pandemie von Unternehmensaufgaben und Insolvenzen, Unternehmenszusammenschlüssen und verminderten Gründungsanreizen betroffen. Durch den Digitalisierungsschub infolge der Kontaktbeschränkungen ist zudem zu erwarten, dass Digitalmärkte mit Konzentrationstendenzen an Bedeutung gewinnen. Nachhaltige Strukturveränderungen mit wettbewerbsbeeinträchtigender Auswirkung sind also zu befürchten, und dies angesichts ohnehin bestehender Trends zu ansteigender Marktmacht und Konzentration in Teilbereichen der Wirtschaft. Bei den wirtschaftspolitischen Reaktionen auf die krisenbedingten Herausforderungen sollte vor diesem Hintergrund angestrebt werden, den Wettbewerb langfristig funktionsfähig zu erhalten. Wenn die Fusions- und Beihilfenkontrolle ohne materiell-rechtliche Abstriche angewendet würde und staatliche Unternehmensbeteiligungen durch wettbewerbsfördernde Maßnahmen flankiert würden, könnte dies gelingen.

## Abstract (en)

Numerous markets are affected during the coronavirus pandemic by corporate closures and insolvencies, mergers and reduced incentives for start-ups. In addition, the surge in digitisation due to policies intended to reduce personal contacts is expected to lead to an increasing significance of digital markets. Sustained structural changes with adverse effects on competition are therefore to be expected against the backdrop of existing trends toward increasing market power and concentration in certain sectors of the economy. With this in mind, the economic policy responses to the challenges posed by the crisis should consider the requirements of long-term competition. Mergers and state aid control without any substantive legal concessions as well as the flanking of state shareholdings in companies with measures to promote competition would contribute to this.

Die Coronavirus-Pandemie beeinträchtigt weltweit das öffentliche und wirtschaftliche Leben stark. Auch in Deutschland hat die Pandemie zu einem drastischen Konjunktureinbruch geführt, der viele Unternehmen vor große Schwierigkeiten stellt und weitreichende Veränderungen von Marktstrukturen und Auswirkungen auf den Wettbewerb erwarten lässt.[1] Diese Veränderungen resultieren aus Unternehmensaufgaben und Insolvenzen, Unternehmenszusammenschlüssen und eingeschränkter Gründungsaktivität sowie aus den wirtschaftspolitischen Maßnahmen, mit denen dem Wirtschaftseinbruch als Folge der Coronavirus-Pandemie begegnet wird. Zu letzteren gehören die Erleichterung von Unternehmenskooperationen und das umfangreiche Gewähren staatlicher Beihilfen.

Marktstrukturen verändern sich in der Krise. Branchenübergreifend ist grundsätzlich davon auszugehen, dass weniger wettbewerbsfähige Anbieter*innen in einer Rezession aus Märkten ausscheiden, da sie den Nachfragerückgang und verschlechterte Finanzierungsmöglichkeiten schwerer kompensieren können (vgl. unter anderem Foster, Grim und Haltiwanger, 2016). Dadurch kann sich die Konzentration in einzelnen Branchen erhöhen. Eine gestiegene Marktkonzentration wird aus Wettbewerbsperspektive zunächst kritisch gesehen, da durch erhöhte Verhaltensspielräume und erleichterte Koordinationsmöglichkeiten der Unternehmen potenziell die Wettbewerbsintensität abnimmt. Gleichzeitig kann ein Ausscheiden von weniger effizienten Wettbewerber*innen aber auch zu einem Anstieg der durchschnittlichen Produktivität führen. Allgemein geht eine abnehmende Wettbewerbsintensität jedoch tendenziell mit geringerem Produktivitätswachstum einher, worauf aktuelle Untersuchungen hindeuten (vgl. SVR, 2019, Kapitel 2; Monopolkommission, 2020, Kapitel II).[2] In diesem Zusammenhang sind insbesondere Strukturveränderungen von digitalen Plattformmärkten genauer zu beobachten. Die weltweiten Kontaktbeschränkungen haben zu einer zunehmenden Nutzung digitaler Dienste geführt, etwa durch vermehrte Videokonferenzen und Online-Einkäufe. Durch diesen pandemiebedingten Digitalisierungsschub ist eine weitere Marktmachtzunahme der großen Digitalunternehmen zu erwarten. In Anbetracht von Konzentrationstendenzen auf digitalen Märkten dürfte die Bedeutung der Missbrauchsaufsicht auf diesem Feld noch wichtiger werden.[3]

1 Der Sachverständigenrat zur Begutachtung der gesamtwirtschaftlichen Entwicklung prognostiziert in seinem aktuellen Jahresgutachten einen Rückgang der Wirtschaftsleistung um 5,1 % im Jahr 2020 (SVR, 2020).

2 Der Zusammenhang von Wettbewerb und Produktivitätswachstum ist auch Gegenstand aktueller Untersuchungen (vgl. unter anderem Ganglmair et al., 2020). Für einen Überblick über die möglichen Folgen einer sinkenden Wettbewerbsintensität, siehe Wambach und Weche (2020).

3 Zuletzt hatte die vom Bundesministerium für Wirtschaft und Energie eingesetzte Kommission Wettbewerbsrecht 4.0 die Einführung einer speziellen Plattformverordnung für marktbeherrschende Online-Plattformen vorgeschlagen (Kommission Wettbewerbsrecht 4.0, 2019). Für weitere Ausführungen zur Problematik der Missbrauchsaufsicht in der Plattformwirtschaft siehe Monopolkommission (2020, Tz. 90 ff.).

Für einen funktionsfähigen Wettbewerb und damit ein wohlstandsmehrendes Wirtschaftssystem sind auch die wirtschafts- und wettbewerbspolitischen Reaktionen auf die pandemiebedingte Wirtschaftskrise vor diesem Hintergrund von hoher Bedeutung. Sie sollten auf ihre Angemessenheit und die zu erwartenden Auswirkungen auf den Wettbewerb hin geprüft werden. Beispielsweise könnten staatliche Beihilfen und Unternehmensbeteiligungen den Wettbewerb in den betroffenen Märkten verzerren.

Um die mittel- bis langfristigen Folgen der Coronavirus-Pandemie für den Wettbewerb abzuschätzen, ist ein Blick auf die drei Marktstrukturdeterminanten Markteintritte, Marktaustritte sowie Unternehmenszusammenschlüsse notwendig. Insgesamt werden die drei nachfolgend skizzierten Mechanismen in vielen von der Krise betroffenen Wirtschaftsbereichen die Unternehmenskonzentration ansteigen lassen. Laut einer Einschätzung des ifo Instituts sind in Deutschland insbesondere bereits die Reisebranche, der Fahrzeugbau, das Gastgewerbe sowie der Kulturbereich betroffen (ifo Institut, 2020).

**Dr. David Benček** ist Analyst im wissenschaftlichen Stab der Monopolkommission.

**Lorela Ceni-Hulek**, LL.M., ist dort Analystin.

**Prof. Achim Wambach, Ph.D.**, ist Präsident des ZEW – Leibniz-Zentrum für Europäische Wirtschaftsforschung in Mannheim und Mitglied der Monopolkommission.

**Prof. Dr. John Weche** ist Professor für Volkswirtschaftslehre und Standortpolitik an der Hochschule für Angewandte Wissenschaften (HAW) Hamburg und Senior Analyst im wissenschaftlichen Stab der Monopolkommission.

## Hohe Zahl von Marktaustritten zu erwarten

Durch den Nachfragerückgang, verschlechterte Finanzierungsmöglichkeiten und die Beeinträchtigung von Lieferketten steigt in der Rezession die Wahrscheinlichkeit, dass Unternehmen aus dem Markt austreten, entweder durch Geschäftsaufgabe oder Insolvenz. Mit zunehmender Dauer der Krise stehen auch immer mehr Großunternehmen vor erheblichen Problemen, besonders gefährdet waren von Beginn an aber kleine und mittlere Unternehmen (KMU) (ZEW, 2020). Bei der Finanzierung spielt die Bonität eine wichtige Rolle und kleinere Unternehmen verfügen tendenziell über schlechtere Bonitätsbewertungen als größere. Falls überwiegend kleinere Unternehmen schließen müssen, wäre ein Anstieg in der Unternehmenskonzentration die Folge. Beispiele für Branchen mit hohen Anteilen kleiner Unternehmen mit schwacher Bonität sind in Deutschland die Gastronomie, Kfz-Zulieferer, die chemische Industrie und das Baugewerbe (ZEW, 2020).

Bisher liegt die Zahl angemeldeter Insolvenzverfahren zwar deutlich unter dem Vorjahreswert, dies ist aber auf die Aussetzung der Insolvenzantragspflicht für Unternehmen bis zum 31. Dezember 2020 zurückzuführen.[4] Für die Zahl der Unternehmen, die ihr Geschäft aufgegeben haben, liegen keine robusten Zahlen vor. Laut dem Institut für Wirtschaftsforschung Halle (2020) ist zu beobachten, dass die Zahl der von Insolvenzen betroffenen Beschäftigten erhöht ist, was auf eine Ausweitung von Zahlungsschwierigkeiten auch für größere Unternehmen hindeutet. Aktuelle Simulationen der Bundesbank lassen einen Anstieg der Insolvenzen um 35 % zum 1. Quartal 2021 erwarten (Bundesbank, 2020, 43/44). Vor allem im Verarbeitenden Gewerbe sei von einem deutlichen Anstieg der Unternehmensinsolvenzen auszugehen, sodass es mit einem Quartalswert von rund 750 zu ähnlich vielen Insolvenzen wie nach der globalen Finanzkrise 2007/2008 kommen könnte. Im Vergleich zum 1. Quartal 2020 wäre dies eine Verdoppelung der Insolvenzen in diesem Sektor.

## Mehr Unternehmenszusammenschlüsse möglich

Der Konjunktureinbruch kann auch zu einem Anstieg der Zahl der Unternehmenszusammenschlüsse führen, insbesondere wenn finanziell angeschlagene Unternehmen zwischen Verkauf und Geschäftsaufgabe wählen müssen. Durch die unmittelbar verminderte Wettbewerberzahl haben Zusammenschlüsse direkte Auswirkungen auf Unternehmenskonzentration und Marktstrukturen. Zuletzt ist die Zahl der Fusionskontrollanmeldungen beim Bundeskartellamt in den Monaten April und Mai 2020 zwar im Vergleich zu den Vorjahresmonaten zurückgegangen. Ähnlich wie bei den Insolvenzanmeldungen ist dieser Rückgang aber wahrscheinlich auf die Verschiebung von Anmeldungen zurückzuführen – um die das Bundeskartellamt Unternehmen ausdrücklich gebeten hatte. In den kommenden Monaten wird mit einer deutlichen Zunahme

4 Nach Angaben des Statistischen Bundesamts lagen die gemeldeten Unternehmensinsolvenzen im August 2020 zuletzt fast 40 % unter dem Wert für August 2019 (Destatis, 2020a).

von Fusionskontrollverfahren gerechnet.[5] In der Finanzkrise 2007 sind die Zusammenschlussanmeldungen beim Bundeskartellamt im selben Jahr um ca. 25 % gestiegen (Monopolkommission 2020, 154). Eine wichtige Rolle könnten in Krisenzeiten Sanierungsfusionen spielen. Allerdings wurden während der vergangenen Finanz- und Wirtschaftskrise Freigaben mit Verweis auf Sanierungsfusionen nicht vermehrt gewährt (Monopolkommission, 2010, Tz. 329; Monopolkommission, 2012, Tz. 404).

## Abnehmende Gründungsanreize

Schließlich ist davon auszugehen, dass der pandemiebedingte Abschwung auch die Zahl der Unternehmensgründungen negativ beeinflusst. So rechnet mehr als die Hälfte der Industrie- und Handelskammern in Deutschland 2020 mit weniger bzw. deutlich weniger Unternehmensgründungen (DIHK, 2020). Neue Unternehmen im Markt können dazu beitragen, die konzentrationssteigernde Wirkung von Zusammenschlüssen und Marktaustritten zu kompensieren. Gründungen und Markteintritte stellen einen wichtigen Faktor für neue Wettbewerbsimpulse dar.

Allerdings wiesen bereits vor der aktuellen Krise relevante Indikatoren einen negativen Trend bei Gründungen auf. So überstieg die Zahl von Unternehmensliquidationen zuletzt die der Gründungen (IfM, 2020), und auch die Gründungsrate folgt in Deutschland einem langfristig negativen Trend (Eurostat, 2020). Laut wissenschaftlichem Beirat des Bundesministeriums für Wirtschaft und Energie (2020) ist zu befürchten, dass Start-ups durch mangelnde Finanzierungsmöglichkeiten in der Corona-Krise kurzfristig besonders hart getroffen werden und die Gründerkultur in Deutschland nachhaltig Schaden nimmt. Insofern stellt das 2 Mrd. Euro umfassende Hilfspaket der Bundesregierung für Start-ups eine wichtige Maßnahme dar, um den möglichen negativen Effekten für Neugründungen entgegenzuwirken.

## Pandemiebedingter Konzentrationsanstieg

Wie stark sich die Unternehmenskonzentration erhöhen wird, kann mithilfe einer ökonometrischen Analyse geschätzt werden, die den Effekt konjunktureller und konjunkturpolitischer Einflüsse auf Konzentrationskennzahlen untersucht. Dazu muss zunächst der Konjunktureinfluss auf einzelne Wirtschaftsbereiche gemessen werden. Ein naheliegender Indikator wäre der Umsatz- oder Wertschöpfungsrückgang. Allerdings würde dieser zwar die unmittelbare Auswirkung auf die wirtschaftliche Aktivität widerspiegeln, ein solches Maß würde jedoch Markteingriffe des Staates durch Finanzhilfen, Beteiligungen oder regulatorische Erleichterungen nicht vollumfänglich berücksichtigen. Besser geeignet ist deshalb ein Ex-post-Maß auf Basis des Marktergebnisses, das potenziell vorhandene Markteingriffe einschließt. In diesem Sinne stellen Insolvenzraten einen geeigneten Indikator dar, da sich in ihnen Entscheidungen von Unternehmen unter Einbeziehung aller wirtschaftlichen und institutionellen Rahmenbedingungen widerspiegeln und sie somit die Auswirkungen der Konjunkturentwicklung eines Sektors auf seine Marktstruktur abbilden.

Abbildung 1

**Unternehmensinsolvenzen in Deutschland**

Index 2008 = 100

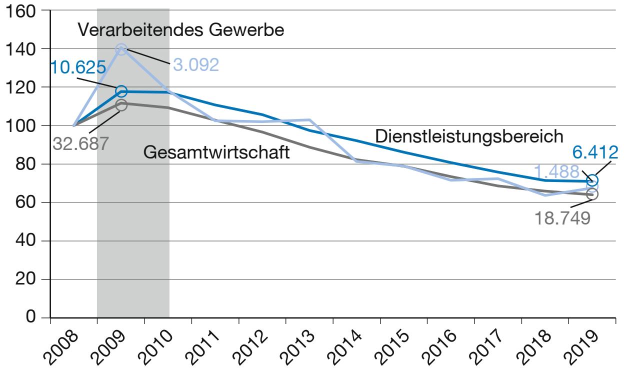

Anmerkungen: Der Krisenzeitraum von Ende 2008 bis Mitte 2010 ist grau eingefärbt. Für jede Zeitreihe ist die absolute Zahl beantragter Insolvenzen im Jahr 2019 sowie zum Höhepunkt der Krise 2009 angegeben.

Quelle: Destatis (2020b); eigene Berechnungen.

In der jüngeren Vergangenheit stellen die Auswirkungen der globalen Wirtschafts- und Finanzkrise einen mit der Coronavirus-Pandemie vergleichbaren Konjunkturschock für die deutsche Wirtschaft dar. Wie Abbildung 1 verdeutlicht, kam es 2009 sowohl gesamtwirtschaftlich als auch in einzelnen Bereichen der Wirtschaft zu einem sprunghaften Anstieg der Unternehmensinsolvenzen. Im Vergleich zur aktuellen Krise unterscheiden sich zwar Ausmaß und Betroffenheit einzelner Wirtschaftszweige, dennoch gibt es auch Gemeinsamkeiten. Beide Krisen entfalten globale Effekte, die z. B. Lieferketten, den internationalen Handel und die Realwirtschaft beeinträchtigen. Gleichwohl gilt es zu berücksichtigen, dass die Kon-

5 Vgl. hierzu unter anderem auch die Einschätzung des Präsidenten des Bundeskartellamts: „Kartellamt rechnet mit Übernahmewelle“, Süddeutsche Zeitung vom 11. Mai 2020. Darüber hinaus wird z. B. auch in der Baubranche sowie dem gesamten Mittelstand mit einer Konsolidierungswelle gerechnet (vgl. unter anderem „In der Bauindustrie geht die Angst vor dem Crash um“, Handelsblatt vom 15. Juni 2020 und „Selbst solide Mittelständler geraten in Existenznot“, Handelsblatt vom 20. April 2020).

junktur infolge der Pandemie wesentlich schneller eingebrochen ist als Ende 2008.

Ein kausaler Effekt des Konjunktureinbruchs auf die Entwicklung der Unternehmenskonzentration kann mithilfe eines Differenz-in-Differenzen-Ansatzes geschätzt werden. In quasiexperimenteller Form werden zwei Gruppen von Wirtschaftszweigen verglichen, die sich annahmegemäß im Durchschnitt einzig darin unterscheiden, ob sie dem besagten Konjunkturschock ausgesetzt waren oder nicht. Für die vorliegende Analyse wurden deshalb zwei Vergleichsgruppen („hohe Insolvenzrate“ und „niedrige Insolvenzrate“) auf Basis der jeweiligen Insolvenzraten während der Finanzkrise in 4-Steller-Wirtschaftszweigen gebildet.[6] Zur Messung der Unternehmenskonzentration dienen Herfindahl-Hirschman-Indizes (HHI) (Heidorn und Weche, 2020).

Nach der Grundannahme des Differenz-in-Differenzen-Ansatzes folgen beide Gruppen demselben grundlegenden Trend, d. h. die durchschnittliche Unternehmenskonzentration entwickelt sich ohne andere Einflüsse über die Zeit parallel.[7] Der einzige Einfluss – dessen Effektgröße diese Analyse bestimmen soll – ist der Konjunkturschock im Rahmen der Wirtschaftskrise (im grau eingefärbten Bereich von Abbildung 2). Wären die beiden Gruppen gleich stark betroffen gewesen, wäre die Trendlinie der Wirtschaftszweige mit hoher Insolvenzrate dem gepunkteten kontrafaktischen Trend gefolgt. Stattdessen stieg die Unternehmenskonzentration in diesen Wirtschaftszweigen durch krisenbedingte Strukturveränderungen an und lag im Jahr 2011 im Durchschnitt 10 % höher als dies unter sonst gleichen Umständen zu erwarten gewesen wäre.

Falls sich diese Ergebnisse übertragen lassen, ist zu erwarten, dass auch die Coronavirus-Pandemie mit ihren wirtschaftlichen Auswirkungen die Unternehmenskonzentration in stark betroffenen Wirtschaftszweigen erhöhen wird. Mittelfristig wird in diesen Branchen die Konzentration um durchschnittlich 10 % höher liegen als dies ohne die Pandemie der Fall wäre. Die Auswirkungen einer solchen sprunghaft zunehmenden Unternehmenskonzentration auf die tatsächliche Wettbewerbssituation auf Märkten sind indes unklar. Einerseits erhöhen konzentrierte Branchen das Risiko von Oligopolisierungstendenzen. Andererseits können Konzentrationssteigerungen auch einhergehen mit einer Intensivierung des Wettbewerbs unter den verbleibenden Marktteilnehmenden, etwa wenn es sich bei diesen um die besonders produktiven Unternehmen handelt.

Abbildung 2

**Durchschnittliche Unternehmenskonzentration**

4-Steller-Wirtschaftszweige mit hohen und geringen Insolvenzraten, Herfindahl-Hirschman-Index

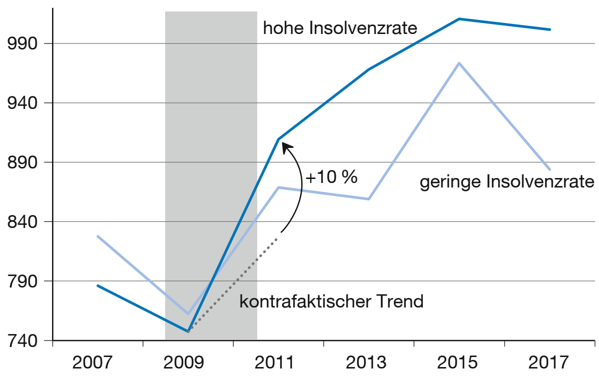

Anmerkung: Der Krisenzeitraum von Ende 2008 bis Mitte 2010 ist grau eingefärbt. Abgetragene Werte entsprechen dem umsatzgewichteten Durchschnittswert der 4-Steller-Wirtschaftszweige in der jeweiligen Gruppe. Die gepunktete Linie bildet die kontrafaktische Entwicklung der durchschnittlichen Unternehmenskonzentration in Wirtschaftszweigen mit hohen Insolvenzraten ab, wenn es keine Wirtschaftskrise gegeben hätte.

Quelle: Destatis (2020b); Heidorn und Weche (2020); eigene Berechnungen.

## Europäische Beihilfenkontrolle während der Coronavirus-Pandemie angemessen

Die Bundesregierung hat auf die pandemiebedingten Herausforderungen sehr schnell reagiert, indem sie zur Abmilderung der sozioökonomischen Folgen der gegenwärtigen Krise ein mehrere hundert Milliarden Euro starkes Maßnahmenpaket auf den Weg gebracht hat (BMWi, 2020). Neben unternehmensunspezifischen Maßnahmen zur Abwendung pandemiebedingter Marktaustritte, ungewollter Unternehmensübernahmen und zur Verbesserung von Gründungsanreizen werden auch gezielt einzelne Unternehmen unterstützt, was zu Wettbewerbsverzerrungen

6 Zwar ist es fraglich, ob einzelne Wirtschaftszweige in der Kontrollgruppe überhaupt nicht von der Konjunkturentwicklung während der Finanz- und Wirtschaftskrise betroffen waren. Für eine sachgerechte Analyse ist es jedoch ausreichend, die Unterscheidung anhand der Stärke des jeweils erlittenen Konjunkturschocks vorzunehmen. Deshalb können die beiden Vergleichsgruppen auf Basis der jeweiligen Insolvenzraten in 4-Steller-Wirtschaftszweigen gebildet werden. Zur Bestimmung der Insolvenzraten wurden die absoluten Insolvenzen der Krisenjahre 2009 und 2010 je 4-Steller-Wirtschaftszweig aufsummiert und in Relation gesetzt zur Anzahl der aktiven Unternehmen vor Krisenbeginn im Jahr 2007. Die Gruppenzuordnung basiert auf der Verteilung dieser Insolvenzraten: Die mittleren 10 % zwischen dem 45. und 55. Perzentil wurden von der Analyse ausgeschlossen; der obere Teil der Verteilung bildet die Gruppe „hohe Insolvenzrate“, der untere Teil die Gruppe „geringe Insolvenzrate“.

7 Die Parallelität der beiden Trends in Abbildung 2 wird durch den Ausreißer der Gruppe „geringe Insolvenzrate“ 2013 durchbrochen, längerfristig folgen beide Linien demselben Trend. Die Konzentrationsdaten liegen nicht über einen ausreichend langen Zeitraum vor, wie sie für umfangreichere ökonometrische Tests erforderlich wären.

führen kann. Auch in der Krise greift die EU-Beihilfenkontrolle. Die Mehrzahl der staatlichen Maßnahmen zur Unterstützung von Unternehmen muss bei der Europäischen Kommission angemeldet und darf nicht ohne deren Genehmigung umgesetzt werden.

Um die Maßnahmen der EU-Mitgliedstaaten zu erleichtern, hat die Europäische Kommission bereits früh, am 19. März 2020, in ihrer Mitteilung über einen „Befristeten Beihilferahmen“ die Voraussetzungen dargelegt, die erfüllt sein müssen, damit staatliche Maßnahmen zur Unterstützung der Unternehmen in der Coronavirus-Pandemie als EU-beihilferechtskonform betrachtet werden können (EU-Kommission, 2020a, 5 ff., Tz. 22 ff). Der Befristete Beihilferahmen soll einen gewissen Spielraum verschaffen, damit jeder EU-Mitgliedstaat seine nationale Wirtschaft in der Corona-Pandemie stützen kann (EU-Kommission, 2020d). Die Europäische Kommission konnte bei der Erarbeitung des Befristeten Beihilferahmens die Erfahrungen aus der Finanzkrise in den Jahren 2008/2009 nutzen. Auch damals hatte sie Mitteilungen erlassen, die mehrfach an die damalige Lage angepasst wurden, um Banken und Unternehmen den Zugang zu Finanzierungsmitteln zu erleichtern (EU-Kommission, 2020e).

Auf Grundlage des Befristeten Beihilferahmens konnten die krisenbedingten Hilfsmaßnahmen der EU-Mitgliedstaaten meist schnell durch die Europäische Kommission genehmigt werden. Die Genehmigung des deutschen Wirtschaftsstabilisierungsfonds (WSF) benötigte hingegen mehr Zeit – das Gesetz zur Errichtung des WSF wurde am 24. März 2020 als Beihilferegelung bei der Europäischen Kommission notifiziert und erst am 8. Juli 2020 von ihr genehmigt.[8] Der Befristete Beihilferahmen wurde viermal erweitert und um zusätzliche Unterstützungsmaßnahmen ergänzt. Zuletzt beschloss die Europäische Kommission, den Befristeten Beihilferahmen bis zum 30. Juni 2020 und die Bestimmungen zu Rekapitalisierungsmaßnahmen bis zum 30. September 2021 zu verlängern sowie zu erweitern, um Unternehmen mit erheblichen Umsatzeinbußen stärker zu unterstützen (EU-Kommission, 2020h). Vor dieser Erweiterung hatten die EU-Mitgliedstaaten die Möglichkeit, zu dem von der Europäischen Kommission vorgelegten Vorschlagsentwurf Stellung zu nehmen (Beck-aktuell, 2020). Insbesondere ging es im Befristeten Beihilferahmen vom 19. März 2020 um die Bewilligung von direkten Zuschüssen, rückzahlbaren Vorschüssen oder Steuervorteilen für Unternehmen, unter der Bedingung, dass sie wegen der Pandemie, d.h. nach dem 31. Dezember 2019, in Schwierigkeiten geraten sind, und dass die Höhe der Unterstützung 800.000 Euro pro Unternehmen nicht überschreitet. Ferner wurden im Rahmen der ersten Erweiterung vom 3. April 2020 Maßnahmen verabschiedet, wie etwa direkte Zuschüsse, rückzahlbare Vorschüsse und Steuervorteile für Forschung und Entwicklung, den Auf- und Ausbau von Erprobungseinrichtungen für das Coronavirus betreffende Produkte sowie für die Schaffung zusätzlicher Produktionskapazitäten zur Herstellung von für die Krisenbewältigung notwendigen Produkten (EU-Kommission, 2020b).

Insgesamt lässt sich festhalten, dass die Europäische Kommission weitgehend schnell und flexibel auf die Herausforderungen der Coronavirus-Pandemie reagiert hat. Allerdings ist die verzögerte Genehmigung des deutschen Wirtschaftsstabilisierungsfonds unglücklich. Das in diesem Zusammenhang vorgebrachte Argument, dass die Höhe der zur Verfügung stehenden Unterstützung in Deutschland grundsätzlich zu einer Wettbewerbsverzerrung führe, überzeugt nicht. Zum einen ist davon auszugehen, dass manche für Deutschland aufgelegten Programme nicht ausgeschöpft werden, während Programme anderer Länder bei Bedarf aufgestockt werden können. Darüber hinaus werden nur Unternehmen gefördert, die bis Ende 2019 nicht in finanzielle Schwierigkeiten geraten sind und die wahrscheinlich auch nach der Krise erfolgreich sein werden. Ein kleinerer Wirtschaftsstabilisierungsfonds, der nicht für alle Unternehmen, die Liquiditätsunterstützung benötigen und diese Kriterien erfüllen, zur Verfügung stünde, hätte voraussichtlich vermehrte Marktaustritte zur Folge. Dieser Umstand würde keine wettbewerblichen Vorteile bedeuten.

## Voraussetzungen und Auflagen für Staatsbeteiligungen notwendig

In der am 8. Mai 2020 von der Europäischen Kommission verabschiedeten zweiten Erweiterung des Befristeten Beihilferahmens werden unter anderem die Bedingungen dargelegt, unter denen die EU-Mitgliedstaaten den Unternehmen Eigenkapital oder sogenanntes Hybridkapital gewähren können, um Insolvenzen zu vermeiden (EU-Kommission, 2020c). Im Vordergrund dieser Bedingungen steht die Sicherstellung eines wirksamen Wettbewerbs. EU-Mitgliedstaaten müssen zusätzliche Vorkehrungen treffen, wenn der oder die Empfänger*in auf mindestens einem relevanten Markt über beträchtliche Marktmacht verfügt und die Maßnahme mehr als 250 Mio. Euro beträgt. Zudem ist für Empfänger*innen von krisenbedingten Rekapitalisierungsmaßnahmen keine Beteiligung von mehr als 10 % an Konkurrenzbetrieben oder anderen Unternehmen im selben Geschäftsfeld, mitsamt vor- und nachgelagerten Geschäftstätigkeiten, erlaubt, bis nicht mindestens 75 % der Maßnahme zurückgekauft und ver-

8 Einen Überblick über den Stand der bereits genehmigten nationalen Unterstützungsmaßnahmen bietet die Europäische Kommission auf ihrer Internetseite (EU-Kommission, 2020f).

kauft worden sind (EU-Kommission, 2020c). Darüber hinaus dürfen Staatsbeteiligungen an Unternehmen nicht das für die Sicherstellung der Empfänger-Rentabilität notwendige Minimum übersteigen und sollen darauf abzielen, dass die vor der Coronavirus-Pandemie bestehende Kapitalstruktur wiederaufgebaut wird.

### Der Fall Lufthansa

Einer der ersten Fälle von Eigenkapitalerhöhung, der von der Europäischen Kommission am 25. Juni 2020 genehmigt wurde, war der Einstieg des Bundes bei der Deutschen Lufthansa AG. Die Europäische Kommission hat der Beteiligung des deutschen Staates an der Lufthansa unter Auflagen zugestimmt. So müssen 24 Start- und Landerechte an den Drehkreuzen der Lufthansa an den Flughäfen Frankfurt und München und bis zu vier Flugzeuge an Konkurrenzunternehmen übertragen werden. Da eine Staatsbeteiligung an einem Unternehmen zu schwerwiegenderen Wettbewerbsverfälschungen führen kann als Kredite oder staatliche Garantien, dürfe sie nach Ansicht der Europäischen Kommission nur unter strengen Voraussetzungen genehmigt werden.

Die deutsche Monopolkommission (2017, Tz. 213 ff.) erachtet diese Argumentation der Europäischen Kommission als triftig, denn der Staat als Miteigentümer verschafft dem betroffenen Unternehmen unter anderem aufgrund seiner Bonität Vorteile bei Kreditaufnahmen, was ungleiche Wettbewerbsbedingungen zulasten von Wettbewerber*innen ohne staatliche Beteiligung schaffen kann. Eine Einschränkung des Wettbewerbs auf bestimmten Flugstrecken in Deutschland ist spätestens nach der Insolvenz der Air Berlin im Jahr 2017 zu beobachten. So sind auf einigen Strecken innerhalb Deutschlands und Europas die Deutsche Lufthansa und ihre Tochterunternehmen Eurowings, Swiss, Austrian Airlines und Brussels Airlines alleinige Anbieterinnen. Die Abgabe von Start- und Landerechten an Wettbewerber*innen, entweder direkt durch die Fluggesellschaft oder nach Rückgabe an den Staat durch eine Versteigerung an andere Fluggesellschaften, kann somit zu mehr Wettbewerb auf solchen Strecken beitragen (Haucap und Wambach, 2020; Monopolkommission, 2017, Tz. 213 ff.).

### Der Fall Curevac

Ein weiterer Fall einer Staatsbeteiligung ist der Einstieg des Bundes mit 300 Mio. Euro beim Biopharmaunternehmen Curevac. Damit hält der Bund über die Kreditanstalt für Wiederaufbau (KfW) einen Anteil von derzeit etwa 17 % an dem Tübinger Impfstoff-Entwickler (Lambrecht und Baars, 2020). Diese Beteiligung wird seitens der Bundesregierung als „strategische Investition“ bezeichnet, eine kurzfristige Veräußerung sei nicht geplant (Dostert, 2020). Auch wenn in der Öffentlichkeit häufig positiv herausgestellt wird, dass diese Beteiligung (derzeit) betriebswirtschaftlich profitabel ist, so sind Gewinn und Verlust nicht das Maß für den Erfolg einer Staatsbeteiligung (Dostert, 2020). Der Zweck einer Staatsbeteiligung sollte klar erkennbar sein. Gerade in Pandemiezeiten stellt die Sicherung von Impfstofflieferungen für die Bundesregierung ein wichtiges Ziel dar. Beispielsweise hat der Bund ein Sonderforschungsprogramm in Höhe von 750 Mio. Euro aufgelegt. Daraus erhält das Biotechnologie-Unternehmen Biontech bis zu 375 Mio. Euro und Curevac bis zu 252 Mio. Euro, um die laufenden klinischen Studien zu unterstützen und die Produktionskapazitäten auszubauen (Lambrecht und Baars, 2020). Warum eine zusätzliche Beteiligung an einem Unternehmen nötig war, ist nicht ersichtlich. Weitere und weniger wettbewerbsverzerrende Instrumente, wie etwa eine Kaufzusage für Impfstoffe, hätten zum Erreichen des Versorgungsziels zur Verfügung gestanden.

Der Staatseinstieg mit Eigenkapital bei einem Unternehmen sollte gemäß der zweiten Erweiterung des Befristeten Beihilferahmens nur dann in Erwägung gezogen werden, wenn „keine andere geeignete Lösung gefunden werden kann“ (EU-Kommission, 2020c, Rz. 7). Betrachtet man den Fall Curevac näher, deutet der nach dem Staatseinstieg verwirklichte erfolgreiche Börsengang des Unternehmens darauf hin, dass es durchaus andere Möglichkeiten für eine Kapitalbeschaffung gegeben hätte (Dostert, 2020). Und falls es ein industriepolitisches Ziel ist, die Finanzierungsmöglichkeiten für die Biotechbranche insgesamt zu verbessern, so wird dies eher nicht mit der selektiven Finanzierung eines einzelnen Unternehmens erreicht (Dostert, 2020).

Gemäß dem Befristeten Beihilferahmen sollen außerdem eindeutige Voraussetzungen für die Staatsbeteiligung an den betreffenden Unternehmen geschaffen werden: eine angemessene Vergütung des Staates für seine Investition, Governance-Bestimmungen etwa im Hinblick auf Geschäftsexpansionen und das Eingehen von unternehmerischen Risiken sowie geeignete Maßnahmen zur Einschränkung von Wettbewerbsverfälschungen (EU-Kommission, 2020c, Rz. 45). In Anbetracht der Tatsache, dass eine Staatsbeteiligung an Unternehmen ein Kriseninstrument darstellt, sollte sie beendet werden, sobald die aktuelle Krise überwunden ist und sich die Wirtschaft stabilisiert hat. Dies bedeutet vor allem, dass der Bund auch im konkreten Fall Anreize für einen Rück- und Verkauf seiner Anteile schaffen und einen schlüssigen Ausstiegsplan haben sollte, um das Risiko für etwaige Wettbewerbsverzerrungen auf ein Minimum zu beschränken.

### Der Fall Deutsche Bahn

Die Deutsche Bahn ist bereits vollständig im Besitz des Bundes. Geplant ist hier jedoch eine Eigenkapitalerhöhung von 5 Mrd. Euro, welcher der Bundestag bereits zugestimmt hat. Auch wenn der Finanzbedarf des Unternehmens beträchtlich ist, wird diese Hilfsmaßnahme vor allem deshalb kritisiert, weil dadurch Konkurrent*innen der Deutschen Bahn im Wettbewerb benachteiligt werden könnten. Eine Genehmigung der EU-Kommission steht aus.

In ihrem aktuellen Hauptgutachten merkt die Monopolkommission an, dass der Wettbewerb im Bahnverkehr in Deutschland ohnehin schwach ausgeprägt ist und weiter geschwächt würde, wenn die Deutsche Bahn die Hilfe durch den Bund dazu nutzen würde, ihre eigene Wettbewerbsposition als Transportunternehmen zu stärken. Dies könnte vermieden werden, wenn die Unterstützung für die Deutsche Bahn vorrangig zweckgebunden als Investition in das Schienennetz als Infrastruktur fließen würde. Von Investitionen in die von allen Wettbewerber*innen genutzte Infrastruktur würden sowohl die Deutsche Bahn als auch die Konkurrenz profitieren. Soweit die Transportsparten unterstützt werden, sollten diese den Anforderungen der Beihilfenkontrolle entsprechen und gegebenenfalls wettbewerbsunterstützende Vorkehrungen getroffen werden.

### Beihilfen für Start-ups zielführend

In der dritten Erweiterung des Befristeten Beihilferahmens der Europäischen Kommission vom 29. Juni 2020 wurde festgelegt, dass die EU-Mitgliedstaaten kleine und Kleinstunternehmen unterstützen können, auch wenn diese schon am 31. Dezember 2019 in finanzielle Schwierigkeiten geraten waren. Dies wird damit begründet, dass solche Unternehmen[9] besonders von pandemiebedingten Liquiditätsengpässen betroffen sind. Dadurch könnte es zu einem überproportionalen Anstieg von Insolvenzen kommen, der eine ernste Beeinträchtigung der EU-Wirtschaft zur Folge hätte (EU-Kommission, 2020g). Auch Start-ups sollen stärker unterstützt werden. Ausgenommen vom Anwendungsbereich der dritten Erweiterung sind Unternehmen, die Gegenstand eines Insolvenzverfahrens sind, noch zurückzuzahlende Rettungsbeihilfen erhalten haben oder einem Umstrukturierungsplan unter Beachtung der Beihilfevorschriften unterliegen. Darüber hinaus sollen Anreize für private Investierende erhöht werden, sich an coronabedingten Rekapitalisierungsmaßnahmen zu beteiligen. Entscheidet der Staat, eine Rekapitalisierungsbeihilfe anzubieten, bei der die Beteiligung privater Investierender an der Kapitalerhöhung mindestens 30 % des neu zugeführten Kapitals ausmacht und zu den gleichen Bedingungen wie die Staatsbeteiligung geschieht, dann sind laut dritter Erweiterung das Übernahmeverbot und die Vergütungsbeschränkungen der Geschäftsleitung auf drei Jahre befristet. Sofern die Kapitalanteile der Inhaber*innen der bestehenden Anteile zusammen genommen weniger als 10 % betragen, wird das Dividendenverbot für die Inhaber*innen der neuen Anteile und für diejenigen der bestehenden Anteile aufgehoben (EU-Kommission, 2020g).

Wie die Europäische Kommission betont, sollen innovative Unternehmen unterstützt werden, da sie für die wirtschaftliche Erholung der Europäischen Union von wesentlicher Bedeutung seien (EU-Kommission, 2020g). Außerdem würde eine Insolvenzwelle von jungen Unternehmen mittelfristig auch das Interesse an Neugründungen verringern, mit der Folge, dass die wettbewerblichen Impulse durch Start-ups verloren gingen. Befristete Beihilfen für kleine Unternehmen werden aufgrund von deren geringer Beteiligung an grenzüberschreitenden Geschäften und ihrer geringen Marktmacht den Wettbewerb im Binnenmarkt möglicherweise weniger beeinträchtigen (EU-Kommission, 2020g).

### Fusionskontrolle

Angesichts der drohenden Insolvenz- und Übernahmewelle ist aus Sicht der Monopolkommission eine gelockerte Durchführung von Unternehmensübernahmen, etwa durch eine großzügigere Handhabung des fusionskontrollrechtlichen Eingriffskriteriums, dennoch nicht empfehlenswert (Monopolkommission, 2020a).

In Anbetracht der gegenwärtigen Krise könnte das Instrument der Sanierungsfusion (*failing company defence*) wieder zunehmend Beachtung finden. Damit eine Sanierungsfusion vom Bundeskartellamt freigegeben werden kann, müssen die am Zusammenschluss beteiligten Unternehmen nachweisen, dass bestimmte Voraussetzungen kumulativ vorliegen: *Erstens* muss ohne den Zusammenschluss ein unmittelbarer Marktaustritt des Zielunternehmens drohen. Dies ist normalerweise der Fall, wenn das Zielunternehmen unmittelbar vor einer Geschäftsaufgabe steht oder ein Insolvenzverfahren bereits eingeleitet worden ist. *Zweitens* darf kein alternativer Erwerber zur Verfügung stehen, dessen Zusammenschluss mit dem Zielunternehmen sich weniger schädlich auf den Wettbewerb auswirken würde. *Drittens* muss die Marktstellung des Zielunternehmens auch ohne den Zusammenschluss im Wesentlichen dem Erwerber zuwachsen.

9 Dazu zählen Unternehmen mit weniger als 50 Beschäftigten und einem Jahresumsatz und/oder einer Jahresbilanzsumme von weniger als 10 Mio. Euro.

Aufgrund der sehr hohen Anforderungen an die Darlegung und den Nachweis der bereits skizzierten Voraussetzungen gilt eine Sanierungsfusion allerdings eher als eine Ausnahme in der fusionskontrollrechtlichen Praxis. Vor diesem Hintergrund werden in der gegenwärtigen Krisensituation wieder Erwägungen angestellt, die Anforderungen abzusenken. Einerseits machen das sich durch die Krise verschlechternde wirtschaftliche Umfeld und das entsprechende Dringlichkeitsbewusstsein den Marktaustritt von einigen Unternehmen wahrscheinlich (Fountoukakos, Barraud und Barrio, 2020). Andererseits aber könnte es vermehrt zu Fällen kommen, bei denen marktmächtige Wettbewerber*innen unter dem Schirm der durch die Krise ausgelösten finanziellen Schwierigkeiten geschwächte Unternehmen kaufen, was zu weniger Wettbewerb führen kann. Erleichterte Kriterien bei der Sanierungsfusion wurden auch während der Finanzkrise in den Jahren 2008/2009 erwogen. Sie wurden jedoch von Wettbewerbsbehörden mit der Begründung abgelehnt, dass auch das Insolvenz- und Beihilferecht geeignete Instrumente zur Unterstützung von Unternehmen, die von der Krise besonders stark betroffen sind, bereitstellt (OECD, 2009, 12-13).

Zusammenfassend ist davon abzuraten, die Kriterien von Sanierungsfusionen bzw. die fusionskontrollrechtlichen Eingriffskriterien im Allgemeinen mit dem Ziel abzuschwächen, zusätzliche Unternehmenszusammenschlüsse gestatten zu können, insoweit dadurch auch Fusionen zwischen Unternehmen gebilligt werden, die ansonsten nicht aus dem Markt austreten würden. Die Marktstrukturveränderungen und die damit verbundene Abnahme der Wettbewerbsintensität würden auf Dauer bestehen bleiben und könnten nach dem Ende der gegenwärtigen Krise nicht wieder rückgängig gemacht werden.

## Fazit: Funktionsfähigen Wettbewerb nach der Krise im Auge behalten

Vor dem Hintergrund der Coronavirus-Krise ist davon auszugehen, dass der konjunkturelle Einbruch in einzelnen stark betroffenen Wirtschaftszweigen mittelfristig zu einem Anstieg der Unternehmenskonzentration führen wird. Vergleichbare wirtschaftliche Entwicklungen im Zuge der Finanz- und Wirtschaftskrise der Jahre 2008 bis 2010 haben die Konzentration der besonders betroffenen Wirtschaftszweige um durchschnittlich 10 % erhöht. Unklar bleibt jedoch, wie sich ein derartiger Konzentrationsanstieg auf die tatsächliche Wettbewerbssituation auf einzelnen Märkten auswirkt, da sich auch die überdurchschnittlich produktiven und wettbewerbsfähigen Unternehmen am Markt durchsetzen könnten.

Angesichts der krisenbedingten Herausforderungen wurde eine Reihe von wirtschafts- und wettbewerbspolitischen Maßnahmen auf nationaler wie auch auf europäischer Ebene verabschiedet, um in finanzielle Schwierigkeiten geratene Unternehmen zu unterstützen. Dabei weist die Europäische Kommission durch den Erlass des Befristeten Beihilferahmens und dessen Erweiterungen auf die Notwendigkeit von wettbewerbsfördernden Maßnahmen hin, um möglichen Wettbewerbsverzerrungen durch Unterstützung einzelner Unternehmen vorzubeugen. Auch in der Krise tragen die Beihilfen- und Fusionskontrollen zur Sicherstellung eines funktionierenden Wettbewerbs bei, der von ausschlaggebender Bedeutung für die Wirtschaftsstabilisierung und die Verbraucherwohlfahrt ist. Insbesondere sehen die Kriterien des Befristeten Beihilferahmens vor, dass Staatsbeteiligungen an Unternehmen nur zur Bewältigung der Krise in Betracht gezogen und mit einem klaren Ausstiegsplan versehen werden.

Der massive Wirtschaftseinbruch durch die Corona-Pandemie wird zu Veränderungen der Marktstrukturen und Wettbewerbsbedingungen in vielen Bereichen der Wirtschaft führen. Damit auch langfristig ein funktionsfähiger Wettbewerb gewährleistet werden kann, ist darauf zu achten, dass die Instrumente zu dessen Schutz auch in der Krise angewendet und notwendige Hilfsmaßnahmen nach der Krise zeitnah beendet werden.

**Title:** ***Competition in Times of a Pandemic***

**JEL Classification:** *L1, L4, L5, D4*
